# High-Resolution Core Gene-Associated Multiple Nucleotide Polymorphism (cgMNP) Markers for Strain Identification in the Wine Cap Mushroom *Stropharia rugosoannulata*

**DOI:** 10.3390/microorganisms13071685

**Published:** 2025-07-17

**Authors:** Fei Liu, Bin Cao, Hongmei Dai, Guojie Li, Shoumian Li, Wei Gao, Ruilin Zhao

**Affiliations:** 1 State Key Laboratory of Microbial Diversity and Innovative Utilization, Institute of Microbiology, Chinese Academy of Sciences, Beijing 100101, China; liuf@im.ac.cn (F.L.); caob@im.ac.cn (B.C.); daihongmei13@163.com (H.D.); 2 College of Horticulture, Hebei Agricultural University, Baoding 071001, China; liguojie@hebau.edu.cn (G.L.); yylsm@hebau.edu.cn (S.L.); 3 Institute of Agricultural Resources and Regional Planning, Chinese Academy of Agricultural Sciences, Beijing 100081, China; 4 College of Life Sciences, University of Chinese Academy of Sciences, Beijing 100049, China

**Keywords:** strain identification, core gene-associated MNP markers, phylogenetic tree, phylogenomics

## Abstract

*Stropharia rugosoannulata*, an ecologically valuable and economically important edible mushroom, faces challenges in strain-level identification and breeding due to limited genomic resources and the lack of high-resolution molecular markers. In this study, we generated high-quality genomic data for 105 *S. rugosoannulata* strains and identified over 2.7 million SNPs, unveiling substantial genetic diversity within the species. Using core gene-associated multiple nucleotide polymorphism (cgMNP) markers, we developed an efficient and transferable framework for strain discrimination. The analysis revealed pronounced genetic differentiation among cultivars, clustering them into two distinct phylogenetic groups. Nucleotide diversity (π) across 83 core genes varied significantly, highlighting both highly conserved loci under purifying selection and highly variable loci potentially associated with adaptive evolution. Phylogenetic analysis of the most variable gene, Phosphatidate cytidylyltransferase mitochondrial, identified 865 SNPs, enabling precise differentiation of all 85 cultivars. Our findings underscore the utility of cgMNP markers in addressing challenges posed by horizontal gene transfer and phylogenetic noise, demonstrating their robustness in cross-species applications. By providing insights into genetic diversity, evolutionary dynamics, and marker utility, this study establishes a foundation for advancing breeding programs, conservation strategies, and functional genomics in *S. rugosoannulata*. Furthermore, the adaptability of cgMNP markers offers a universal tool for high-resolution strain identification across diverse fungal taxa, contributing to broader fungal phylogenomics and applied mycology.

## 1. Introduction

*Stropharia rugosoannulata* Farl. ex Murrill, commonly known as the wine cap mushroom, is a fast-growing, resilient, and nutritionally valuable edible fungus [1,2]. With an estimated annual production exceeding 490,000 tons in China (https://bigdata.cefa.org.cn/ accessed on 10 July 2025), it is considered one of the most commercially important edible fungi in China, frequently integrated into recycling agricultural systems and functional food production. First domesticated in Germany in the 1960s, *S. rugosoannulata* was later endorsed by the Food and Agriculture Organization (FAO) as a promising cultivated mushroom for developing countries [3]. Renowned for its adaptability, *S. rugosoannulata* is widely cultivated using agricultural by-products, showcasing a robust capacity for lignocellulose degradation [4]. Studies have demonstrated that this species produces extracellular peroxidases during growth in beech wood microcosms, with manganese-oxidizing peroxidases identified as the primary enzymes responsible for lignin degradation [5]. In addition, it has demonstrated potential in environmental applications, such as wastewater treatment, due to its ligninolytic enzymatic activity [6]. Recently, the genome sequences and transcripts of *S. rugosoannulata* have been determined, providing a foundational resource for further genetic studies [7,8,9,10,11].

Despite its ecological and industrial significance, genetic studies on *S. rugosoannulata* remain limited [12], which hampers efforts in breeding, conservation, and understanding its evolutionary relationships. Accurate strain-level identification is crucial, as strains of the same species often differ markedly in phenotypic traits such as cap color, developmental timing, and degradation efficiency [13,14,15]. Traditional methods based on morphology and biochemistry lack resolution and reproducibility, especially for closely related strains [16]. Although molecular markers—such as single nucleotide polymorphisms (SNPs) and multiple nucleotide polymorphisms (MNPs)—have improved strain differentiation in other fungi [13,17,18], their application in *S. rugosoannulata* remains underdeveloped.

Recent advances in molecular marker technologies, particularly MNP markers, offer promising solutions to these challenges. MNP markers have been successfully employed for strain identification in various fungi, including *Lentinula edodes* [17], *Flammulina filiformis* [18], and *Pleurotus eryngii* [19]. Compared to earlier methods like Simple Sequence Repeats (SSR) markers, MNPs offer superior accuracy and accessibility. For instance, SSR markers, though previously used to identify strains of *Agaricus bisporus* [20], are prone to errors during polymerase chain reactions due to DNA polymerase slippage [21,22]. MNP markers avoid such pitfalls, enabling more reliable genetic analyses. However, conventional MNP-based approaches often generate large datasets, posing analytical challenges.

Core gene-associated MNP (cgMNP) markers overcome these limitations by targeting polymorphisms within conserved, single-copy genes [23]. These markers offer a balance between evolutionary stability and informative variation, enabling high-resolution phylogenetic reconstruction and facilitating strain-level discrimination. Recent studies in *A. bisporus* have demonstrated the effectiveness of cgMNP markers in resolving complex strain relationships and uncovering subtle genetic differences [23]. Derived from conserved genomic regions, cgMNPs are less prone to horizontal gene transfer, ensuring high reliability and transferability [23,24]. By enabling the construction of high-resolution phylogenies, these markers provide valuable insights into strain relationships and comparative genomics, making them a versatile tool in fungal phylogenomics [23,25].

In this study, we applied cgMNP markers to a population of 105 *S. rugosoannulata* strains to investigate strain differentiation and population structure. By integrating cgMNPs with whole-genome resequencing, we constructed a high-resolution phylogenetic framework and assessed genetic diversity. This work extends the use of cgMNPs to a new species, providing a genomic resource to support breeding, conservation, and phylogenetic studies in edible mushrooms.

## 2. Materials and Methods

### 2.1. Sample Collection, DNA Extraction and Genome Resequencing

A total of 105 *Stropharia rugosoannulata* strains (Table 1) were selected to represent broad genetic, geographic, and phenotypic diversity. The collection included 85 cultivated and 20 wild strains obtained from major production regions and native habitats across China. All strains were isolated through tissue culture of fresh fruiting bodies, followed by multiple rounds of sub-culturing on potato dextrose agar (PDA) medium. Axenic purity was confirmed via microscopic examination to ensure the absence of contamination. Mycelial biomass was then harvested for genomic DNA extraction using the CTAB method. DNA quality and concentration were assessed with a NanoDrop spectrophotometer (Thermo Fisher Scientific Inc., Waltham, MA, USA) and further quantified using Qubit fluorometry (Thermo Fisher Scientific Inc., Waltham, MA, USA) to ensure sufficient quality for sequencing. Whole-genome sequencing was conducted on the Illumina NovaSeq 6000 platform (Illumina, Inc., San Diego, CA, USA), generating 150 bp paired-end reads for each strain.

### 2.2. Read Mapping and Genotype Calling

Raw reads were trimmed to remove adapter sequences and low-quality bases using fastp [26]. Processed reads were aligned to the *S. rugosoannulata* reference genome (obtained from NCBI) using the Burrows–Wheeler Aligner (BWA) MEM algorithm (version 0.7.17-r1188) [27], achieving an average genome coverage of 98.1% across strains. The mapping rate, genome depth, and quality of aligned reads were analyzed to ensure uniform data quality across samples. The sequencing statistics of the samples are summarized in Table 1 and Table S1. Variant calling was conducted using the SAMtools v1.10 [8], with separate pipelines for identifying SNPs and MNPs. Variants were filtered based on quality scores (QUAL > 30) and read depth (DP > 10) to minimize false positives. We identified a total of 2,760,421 SNPs, with a subset of 433,344 SNPs exhibiting minor allele frequencies (MAF) > 0.05, which were present in more than half of the strains. A Circos plot was generated using the circlize package (v0.4.16) in R [28] to visualize SNP density, MNP positions, and GC content across the genome.

### 2.3. Core Gene Identification and Marker Development

We used BUSCO v5.2.2 [29] to identify core genes in the *S. rugosoannulata* genome, targeting genes conserved across the fungal lineage. Eighty-three core genes were selected as previously based on criteria of single-copy status and uniform distribution across the genome (Table S2). GO category and KEGG pathway were annotated using eggNOG-mapper v2 for eggNOG 5.0 [30]. We identified 10,143 SNPs associated with these genes to be used as core gene-associated MNP (cgMNP) markers, following approaches previously validated in *Agaricus bisporus* [23].

### 2.4. Phylogenetic Analysis and Statistical Analysis

Neighbor-joining (NJ) trees were constructed based on core gene-associated MNP markers to evaluate the genetic relationships among the *S. rugosoannulata* strains. The phylogenetic tree was visualized in iTOL v6 [31], with color-coded rings indicating strain origin (geographic and cultivation source) and cap color for ease of interpretation. Genetic similarity (GS) between two strains was calculated using the following formula: the number of identical core MNP sequences shared between two strains divided by the total number of core MNP sequences analyzed. Descriptive statistics of nucleotide diversity (π) for each core gene were calculated using VCFtools version 0.1.16 [32] to quantify genetic variation. Nucleotide diversity data were visualized through sliding window analysis (window length: 300 bp, step size: 50 bp), highlighting the top 12 genes with the highest diversity values for comparative analysis.

## 3. Results

### 3.1. Genome Resequencing and Identification of Core Gene-Assicated MNP Markers in Stropharia rugosoannulata

**Figure 1:**
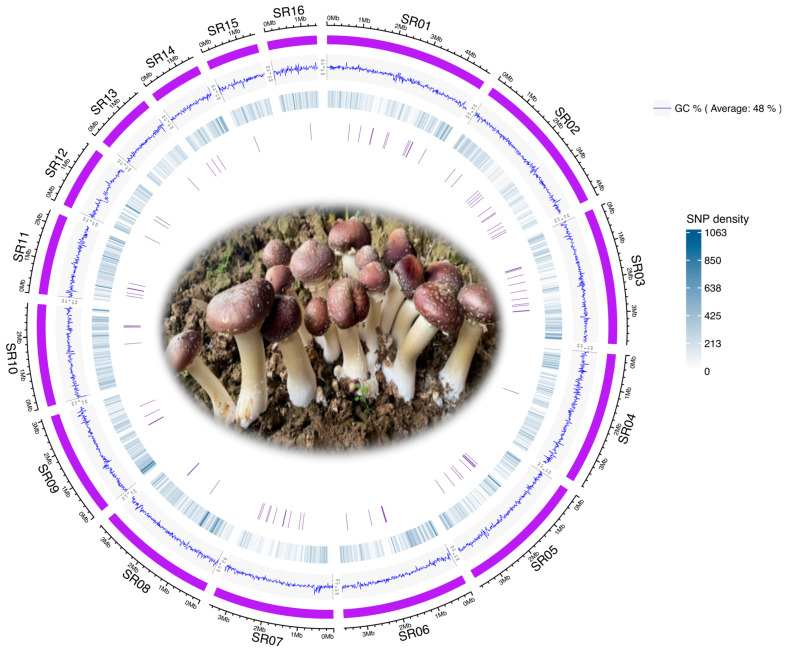
Circos plot of the *Stropharia rugosoannulata* genome, illustrating features from the outermost to innermost circles as follows: (i) the 16 longest scaffolds of the genome, (ii) GC content, (iii) SNP density, and (iv) positions of core gene-associated MNPs.

We collected 105 *Stropharia rugosoannulata* strains, including 20 wild and 85 cultivated strains from diverse geographic locations in China (Table 1). The geographic distribution of all strain collection sites is illustrated in Supplementary Figure S1, created using the *hchinamap* v0.1.0 R package. We sequenced these *S. rugosoannulata* strains, yielding a total of approximately 441.6 Gb of high-quality, clean data, averaging 4.2 Gb per strain (Table S1). Quality metrics were consistently high, with a mean Q20 value of 98.2% and a mean Q30 value of 93.0%, ensuring that the dataset was robust for downstream genetic analyses, including the identification of multiple nucleotide polymorphism (MNP) markers. Mapping reads to the *S. rugosoannulata* reference genome (Figure 1) yielded a mean genome coverage of 98.1%, a mean sequencing depth of 82.3, and an average mapping rate of 96.7% (Table S1). A total of 2,760,421 single nucleotide polymorphisms (SNPs) were identified across the genome. Of these, 433,344 SNPs, with a minor allele frequency (MAF) greater than 0.05 and present in more than half of the 105 strains, were selected as input for the identification of core gene-associated multiple nucleotide polymorphism (cgMNP) markers. A Circos plot illustrates the genome-wide characteristics of *S. rugosoannulata*, showing the locations of MNP markers alongside GC content and SNP density (Figure 1).

To assess the transferability of gene markers initially identified in *Agaricus bisporus*, we examined the presence of 83 core genes in the *S. rugosoannulata* genome using BUSCO. We successfully annotated these genes in the reference genome, identifying a total of 2205 SNPs associated with these core gene markers. The spatial distribution of these MNPs is visualized in Figure 1, demonstrating their genomic distribution and suitability for strain differentiation studies.

### 3.2. Phylogenetic Analysis and Genetic Similarity Values Using Core Gene-Associated MNP Markers

**Figure 2:**
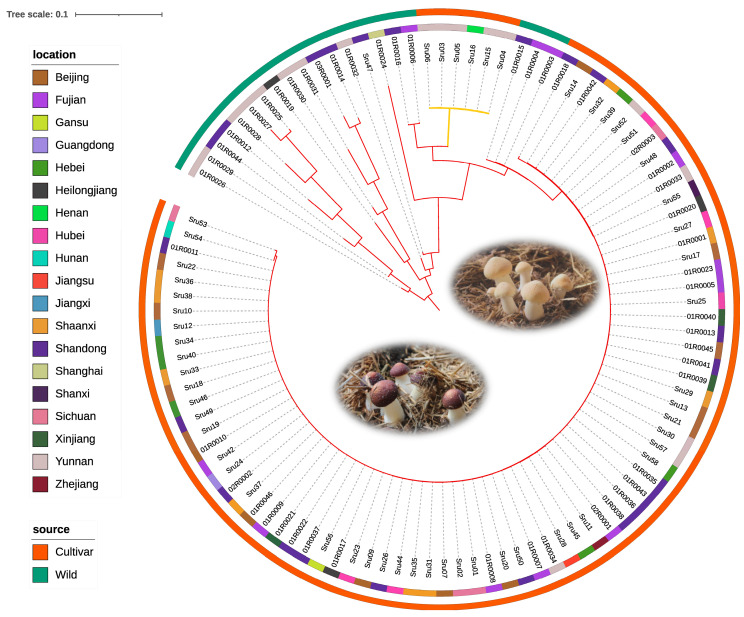
Phylogenetic tree of *Stropharia rugosoannulata* based on core gene-associated MNPs from 105 strains. This neighbor-joining tree, constructed from MNPs within core genes, depicts genetic relationships among wild and cultivated strains of *S. rugosoannulata*, with branch colors representing pileus color (brown/yellow). The innermost ring indicates geographic origin, and the outermost ring distinguishes between wild and cultivated strains, illustrating genetic diversity, geographic distribution, and domestication effects.

Using the 2205 SNPs identified within core gene markers, we constructed a neighbor-joining (NJ) phylogenetic tree to analyze the genetic relationships among the 105 *S. rugosoannulata* strains. This tree revealed distinct genetic subgroups, with patterns of differentiation linked to geographical origin and cultivation status. The NJ tree visualized these relationships, providing insights into evolutionary connections and population dynamics within *S. rugosoannulata* (Figure 2). The analysis supports the utility of core gene-associated MNP markers in exploring complex genetic structures, enabling high-resolution strain identification and evolutionary studies within and beyond *A. bisporus* populations.

**Figure 3:**
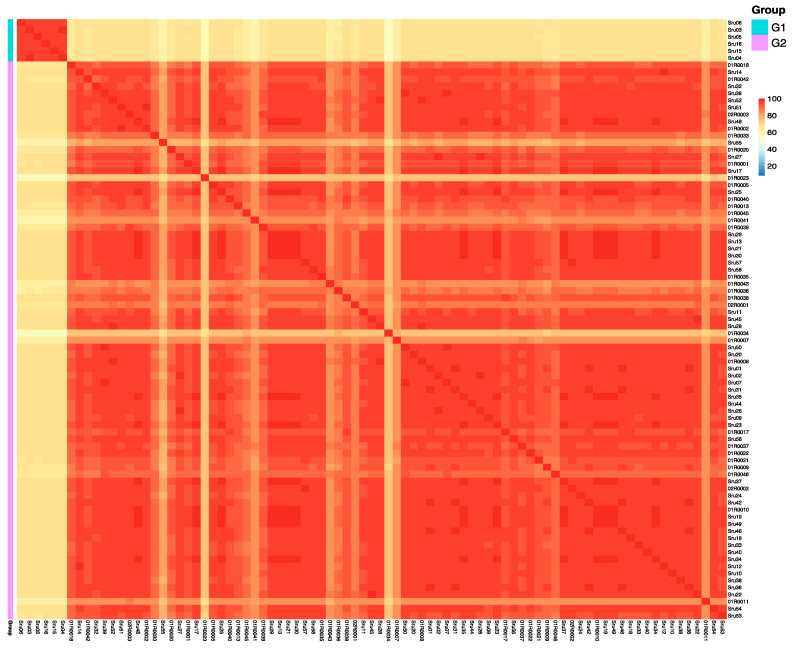
Heatmap illustrating pairwise genetic similarity (GS) values among 85 *Stropharia rugosoannulata* cultivars. Each cell represents the GS value between two strains, with color intensity indicating the degree of genetic similarity. Cultivars are grouped into two distinct pedigrees (G1 and G2) based on a GS threshold of 69.5%, enabling clear separation of genetic lineages. This analysis reveals both intra- and inter-pedigree variation, providing insights into the genetic structure of domesticated *S. rugosoannulata*.

To further explore genetic relationships among the 85 *S. rugosoannulata* cultivars, we calculated genetic similarity (GS) values for all pairwise comparisons. This analysis identified two distinct pedigrees, labeled G1 to G2 (Figure 3; Table S3). The number of strains within each pedigree was as follows: G1 included six strains with yellow pileus, and G2 comprised the majority with 79 strains. GS values within each pedigree exceeded 69.5%, with mean GS values of 96.0% and 91.2%, respectively. A GS threshold of 69.5% effectively differentiated pedigrees, and strains within the same pedigree were considered different cultivars if their GS values ranged from 69.5% to 97.6%.

### 3.3. Nucleotide Diversity Analysis of Core Genes

**Figure 4:**
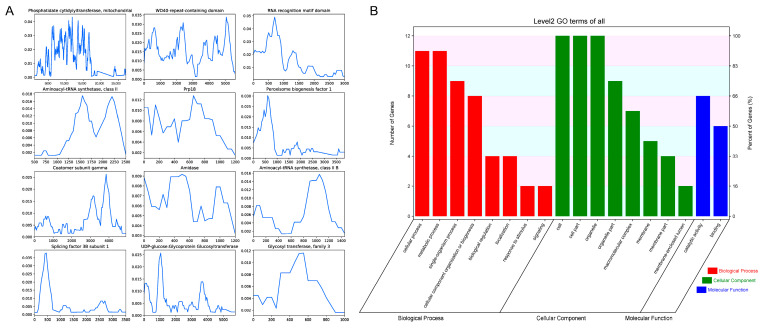
Genetic and functional characterization of the 12 highest diverse genes in *Stropharia rugosoannulata*. (**A**) Nucleotide diversity (π) profiles of the 12 genes with the highest variability, calculated using a sliding window approach (window size: 300 bp; step size: 50 bp). The *Y*-axis represents nucleotide diversity (π), and the *X*-axis denotes relative position along each gene. (**B**) Gene Ontology (GO) functional classification of these 12 genes based on Level 2 annotations. GO terms are grouped into three major categories: biological process, cellular component, and molecular function. The left *Y*-axis indicates the number of genes assigned to each GO category, while the right *Y*-axis shows the proportion of genes within each functional group.

Nucleotide diversity (π) is a crucial metric, as it measures the average degree of polymorphism within a population. A higher π value indicates greater genetic variation, which can be linked to evolutionary adaptation, ecological interactions, and selective pressures. The average value of π across the 83 genes varied between 0.0000 and 0.0160, with a mean π value of 0.0033. Figure 4A presents the nucleotide diversity for the 12 genes exhibiting the highest diversity, which includes genes annotated with “Phosphatidate cytidylyltransferase, mitochondrial,” “WD40-repeat-containing domain,” “RNA recognition motif domain,” “Prp18,” “Coatomer subunit gamma,” “Aminoacyl-tRNA synthetase, class II,” “Glycosyl transferase, family 3,” “Rab-GTPase-TBC domain,” “Actin family,” “Peroxisome biogenesis factor 1,” and “Splicing factor 3B subunit 1.” GO and KEGG analyses revealed that the top 12 highly diverse genes are involved in key functions such as the metabolic process, cellular component organization or biogenesis, organelle, and membrane part (Figure 4B; Table S2).

### 3.4. Phylogenetic Analysis Using the Most Variable Gene

The most variable gene identified in this study is *Phosphatidate cytidylyltransferase mitochondrial* (also known as MMp37 or Tam41), which catalyzes the formation of CDP-diacylglycerol (CDP-DAG) from phosphatidic acid within the mitochondrial inner membrane. CDP-DAG is essential for the biosynthesis of cardiolipin, a dimeric phospholipid that stabilizes supercomplexes of the mitochondrial respiratory chain, thereby playing a critical role in mitochondrial function.

**Figure 5:**
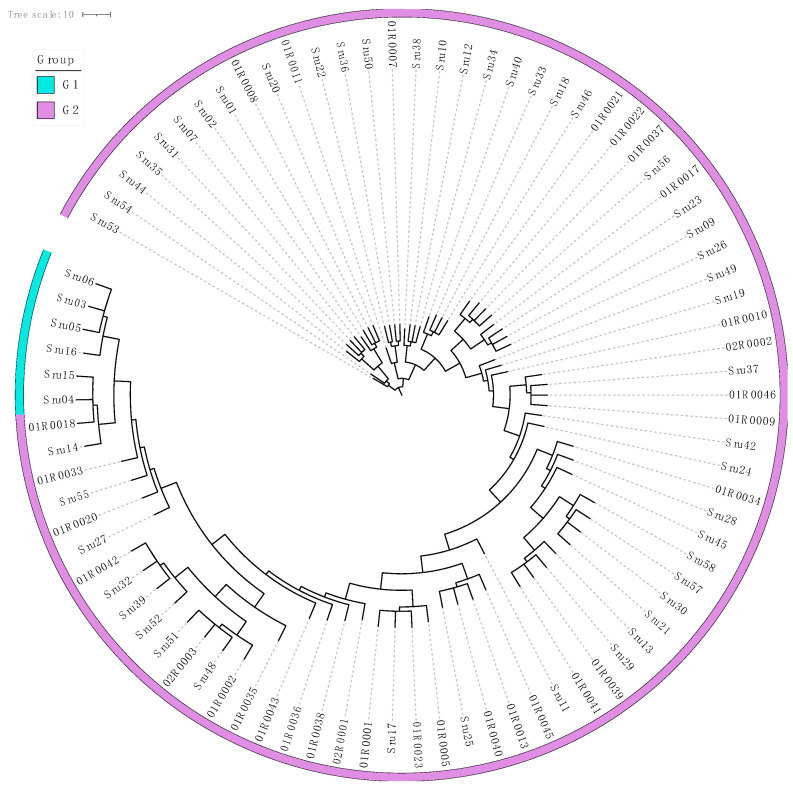
Phylogenetic tree of *Stropharia rugosoannulata* based on the *phosphatidate cytidylyltransferase mitochondrial* gene from 85 cultivars. The phylogenetic tree was constructed using 865 SNPs identified within the *Phosphatidate cytidylyltransferase mitochondrial* gene, demonstrating the genetic relationships among 85 cultivated strains of *S. rugosoannulata*. The outer ring highlights the classification of these strains into two distinct genetic pedigrees (G1–G2), providing clear visualization of their evolutionary divergence. This analysis underscores the utility of this gene as a high-resolution marker for precise strain differentiation and classification.

To further explore its utility as a genetic marker, we selected *Phosphatidate cytidylyltransferase mitochondrial* for phylogenetic analysis of 85 *S. rugosoannulata* cultivars. Leveraging the 865 SNPs identified within this gene, we constructed a phylogenetic tree to assess the genetic relationships among these strains. The analysis demonstrated that this single gene marker could achieve 100% discrimination of all the cultivars (Figure 5). These results highlight the potential of *Phosphatidate cytidylyltransferase mitochondrial* as a robust and precise marker for strain differentiation in *S. rugosoannulata*.

## 4. Discussion

This study provides the first application of core gene-associated MNP (cgMNP) markers in *Stropharia rugosoannulata*, offering new insights into its population structure, domestication patterns, and core gene variability. Resequencing 105 strains revealed over 2.7 million SNPs, and the cgMNP approach enabled high-resolution strain differentiation. Notably, *Phosphatidate cytidylyltransferase mitochondrial* was identified as the most polymorphic gene, serving as a powerful marker for distinguishing cultivars and supporting breeding strategies.

### 4.1. Insights into Stropharia rugosoannulata Genetic Diversity and Phylogeny

Our analysis revealed two major phylogenetic clades, corresponding to wild and cultivated origins. This differentiation likely reflects distinct selective pressures under natural versus managed environments, consistent with patterns observed in other domesticated fungi [12,13]. Among the 83 core genes analyzed, Phosphatidate cytidylyltransferase mitochondrial, involved in lipid metabolism and mitochondrial integrity, exhibited the highest polymorphism, with 865 SNPs. In humans, biallelic variants in *TAMM41* (the human counterpart of this gene) have been linked to reduced cardiolipin levels in muscles, which contribute to neonatal mitochondrial disease [33]. Despite its functional conservation across eukaryotes, this gene alone enabled the complete discrimination of all 85 domesticated strains. Its variability highlights the utility of targeting core functional loci to distinguish closely related genotypes.

Adaptive evolution in *S. rugosoannulata* is likely driven by its exposure to diverse substrates and cultivation systems, leading to selection for traits such as mycelial growth, cap morphology, and lignocellulose degradation. These traits are encoded, in part, by core genes under both purifying and diversifying selection. The cgMNP strategy, by focusing on such genes, effectively captures both deep phylogenetic signals and recent adaptive divergence—making it especially well-suited for studying species under domestication and ecological transition.

While traditional single-gene markers are often limited in resolution [34], cgMNPs leverage polymorphisms within conserved loci to enhance discriminatory power. This genome-informed approach enables precise reconstruction of evolutionary relationships, offering insights into how selection and adaptation have shaped *S. rugosoannulata*’s genetic landscape.

### 4.2. Advantages of Core Gene-Associated MNP Markers

CgMNPs are derived from single-copy core genes that are both functionally essential and taxonomically conserved. This minimizes confounding effects from rapidly evolving or horizontally transferred regions [35,36]. Moreover, cgMNPs strike an effective balance between sequence conservation and informative polymorphism, enabling both broad taxonomic resolution and fine-scale discrimination at the strain level.

Although first applied in *Agaricus bisporus* [23], our study demonstrates the broader applicability of cgMNPs in a phylogenetically distant Basidiomycete. To our knowledge, this is only the second published report utilizing cgMNPs for fungal strain identification, underscoring the novelty of the method. While the formal literature on cross-species transferability remains limited due to the recency of the approach, our findings provide direct and empirical validation: 2205 SNPs derived from these core genes enabled high-resolution phylogenetic reconstruction across 105 strains. These results not only affirm the robustness of cgMNPs but also highlight their promising utility in broader fungal lineages.

This dual capacity of cgMNPs—anchoring analyses in evolutionary conserved loci while still capturing strain-specific variation—is further illustrated by our identification of *Phosphatidate cytidylyltransferase mitochondrial* as the most polymorphic gene. Its 865 SNPs enabled 100% resolution of all 85 cultivars, making it a powerful candidate for rapid strain differentiation.

Beyond this, our analysis of nucleotide diversity (π) across 83 core genes revealed considerable heterogeneity, ranging from highly conserved genes under purifying selection to others with substantial variability—likely reflecting recent adaptation or domestication. Notably, genes containing “WD40-repeat-containing domains” and “RNA recognition motif domains” have well-established roles in eukaryotes, including functions in signal transduction, cell cycle regulation, gene expression, and protein–protein interactions [37,38]. Their high nucleotide diversity in this study suggests adaptive evolution in *S. rugosoannulata*, possibly driven by environmental factors or cultivation conditions. Similarly, the diversity observed in genes such as “Rab-GTPase-TBC domain” and “Coatomer subunit gamma,” which are involved in intracellular transport and vesicle formation, is significant. These processes are crucial for cell growth and development [37]. In contrast, genes exhibiting low diversity likely reflect strong purifying selection, ensuring the maintenance of essential biological functions [12].

These findings lead us to hypothesize that polymorphisms in genes involved in mitochondrial lipid metabolism, RNA processing, and intracellular transport may have been favored during artificial selection. Such adaptations could enhance traits critical to domesticated performance—such as growth rate, stress resilience, and substrate utilization. This framework lays the groundwork for integrating cgMNP-based markers with GWAS and transcriptomics to uncover the functional roles of high-variation loci.

### 4.3. Implications for Breeding and Future Directions

The clear subdivision of strains into genetic groups provides a practical foundation for breeding. These groups facilitate the selection of strains with desirable traits such as yield, stress tolerance, and morphology. The identification of *Phosphatidate cytidylyltransferase mitochondrial* as a highly informative locus supports the integration of gene-specific markers into precision breeding.

In breeding programs, cgMNPs can be used to verify hybrids, trace parentage, and authenticate elite cultivars—supporting intellectual property protection and germplasm management. The 865-SNP profile within a single gene could be adapted into barcode panels or diagnostic kits for rapid in-lab or field-based identification.

Beyond genetic applications, *S. rugosoannulata* has a strong ecological value. Its ability to grow agricultural waste and degrade lignin-rich substrates makes it a valuable species in circular agriculture. Recent studies have underscored the broader agricultural value of edible mushrooms: a livestock–crop–mushroom circular system improves plant growth and reduces antibiotic resistance in soils [39], while spent mushroom substrate enhances microbial abundance and enzymatic activity across farming systems [40]. These findings reinforce the ecological relevance of the genomic markers we developed.

Looking ahead, expanding the core gene set and validating cgMNPs across more fungal taxa will strengthen their applicability. Addressing issues such as horizontal gene transfer and gene rate heterogeneity will further improve phylogenomic precision. Importantly, integrating polymorphic core gene markers into breeding pipelines offers practical advantages. For instance, cgMNP markers can be used to verify hybrid authenticity, trace parentage in controlled crosses, and distinguish elite cultivars in variety registration or field trials. These applications support efficient germplasm selection, protect intellectual property, and accelerate genetic improvement in *S. rugosoannulata* and other edible mushrooms.

## 5. Conclusions

This study demonstrates that core gene-associated MNP (cgMNP) markers enable accurate and high-resolution strain identification in *Stropharia rugosoannulata*. Using over 2.7 million SNPs from 105 strains, we characterized population structure and genetic diversity with precision. A single gene—*Phosphatidate cytidylyltransferase mitochondrial*—proved sufficient to distinguish all cultivars, highlighting the power of gene-specific markers. Beyond this species, cgMNP markers show promise for broader fungal applications due to their balance of evolutionary conservation and polymorphism. These findings offer valuable genomic resources for breeding, conservation, and phylogenomic research in edible mushrooms.

## Figures and Tables

**Table 1:** Basic information of whole-genome sequenced 105 *Stropharia rugosoannulata* strains.

Sample	BaseSum (G)	Mean Depth	Source	Location	Color
01R0001	2.4634890	48.82	Cultivar	Shaanxi	brown
01R0002	2.7003492	53.53	Cultivar	Fujian	brown
01R0003	2.2118484	43.84	Wild	Fujian	brown
01R0004	2.4641238	47.61	Wild	Fujian	brown
01R0005	3.2382552	63.92	Cultivar	Fujian	brown
01R0006	3.1049862	61.25	Wild	Fujian	brown
01R0007	2.1526557	42.72	Cultivar	Fujian	brown
01R0008	2.8159038	55.81	Cultivar	Fujian	brown
01R0009	2.4095355	46.78	Cultivar	Fujian	brown
01R0010	3.8067042	75.28	Cultivar	Beijing	brown
01R0011	2.2150770	43.84	Cultivar	Shandong	brown
01R0012	2.3642259	44.65	Wild	Shandong	brown
01R0013	2.2152048	43.81	Cultivar	Shandong	brown
01R0014	2.7182691	53.96	Wild	Shandong	brown
01R0015	2.3323134	46.26	Wild	Shandong	brown
01R0016	2.1267690	41.98	Wild	Shandong	brown
01R0017	2.3049033	45.76	Cultivar	Heilongjiang	brown
01R0018	2.2960254	45.33	Cultivar	Shandong	brown
01R0019	2.5697190	48.79	Wild	Heilongjiang	brown
01R0020	2.1695034	42.97	Cultivar	Heilongjiang	brown
01R0021	2.5653045	50.58	Cultivar	Xinjiang	brown
01R0022	2.3667072	46.81	Cultivar	Shandong	brown
01R0023	1.8403629	36.23	Cultivar	Fujian	brown
01R0024	2.3851818	45.32	Wild	Shanghai	brown
01R0025	2.3525052	43.29	Wild	Yunnan	brown
01R0026	2.2615755	40.93	Wild	Yunnan	brown
01R0027	2.6118186	48.34	Wild	Yunnan	brown
01R0028	2.3147187	42.99	Wild	Yunnan	brown
01R0029	2.0687826	38.71	Wild	Yunnan	brown
01R0030	2.3813883	43.69	Wild	Yunnan	brown
01R0031	2.1197256	38.65	Wild	Yunnan	brown
01R0032	2.0447976	37.89	Wild	Yunnan	brown
01R0033	2.0854716	41.14	Cultivar	Yunnan	brown
01R0034	2.3866515	47.10	Cultivar	Yunnan	brown
01R0035	2.4101256	47.73	Cultivar	Shandong	brown
01R0036	2.4361248	47.90	Cultivar	Shandong	brown
01R0037	2.4706656	48.73	Cultivar	Shandong	brown
01R0038	2.8203177	55.23	Cultivar	Shandong	brown
01R0039	2.3010948	45.61	Cultivar	Xinjiang	brown
01R0040	2.2662033	44.86	Cultivar	Xinjiang	brown
01R0041	2.0078208	39.72	Cultivar	Shandong	brown
01R0042	2.2996011	45.61	Cultivar	Shandong	brown
01R0043	1.9783281	38.87	Cultivar	Shandong	brown
01R0044	2.2833378	43.88	Wild	Shandong	brown
01R0045	2.1330795	42.21	Cultivar	Beijing	brown
01R0046	2.3766336	46.77	Cultivar	Beijing	brown
02R0001	2.1366321	42.40	Cultivar	Fujian	brown
02R0002	3.9907788	78.80	Cultivar	Shandong	brown
02R0003	4.0328454	78.92	Cultivar	Sichuan	brown
03R0001	2.1084636	40.96	Wild	Shandong	brown
Sru01	5.6994243	113.32	Cultivar	Sichuan	brown
Sru02	5.5662936	111.02	Cultivar	Sichuan	brown
Sru03	5.9468457	118.30	Cultivar	Yunnan	yellow
Sru04	5.8567626	116.58	Cultivar	Yunnan	yellow
Sru05	5.5058730	109.77	Cultivar	Yunnan	yellow
Sru06	5.8558644	116.71	Cultivar	Yunnan	yellow
Sru07	5.7291126	113.94	Cultivar	Beijing	brown
Sru09	5.1560214	102.65	Cultivar	Beijing	brown
Sru10	5.7752538	114.96	Cultivar	Beijing	brown
Sru11	5.6348997	112.48	Cultivar	Zhejiang	brown
Sru12	5.4018870	107.45	Cultivar	Jiangxi	brown
Sru13	5.8063167	115.58	Cultivar	Beijing	brown
Sru14	5.7922959	115.30	Cultivar	Beijing	brown
Sru15	5.8244898	116.15	Cultivar	Yunnan	yellow
Sru16	5.9801625	119.33	Cultivar	Henan	yellow
Sru17	5.7301551	114.16	Cultivar	Beijing	brown
Sru18	5.8504152	116.27	Cultivar	Beijing	brown
Sru19	5.9063601	117.76	Cultivar	Beijing	brown
Sru20	5.9725761	119.05	Cultivar	Beijing	brown
Sru21	5.7092889	113.46	Cultivar	Beijing	brown
Sru22	5.6090259	111.86	Cultivar	Beijing	brown
Sru23	5.8316094	116.25	Cultivar	Hubei	brown
Sru24	5.6767800	112.93	Cultivar	Guangdong	brown
Sru25	5.7882921	115.13	Cultivar	Hubei	brown
Sru26	5.9944332	119.12	Cultivar	Shandong	brown
Sru27	5.9446413	118.46	Cultivar	Hubei	brown
Sru28	5.5093176	109.88	Cultivar	Jiangsu	brown
Sru29	5.7809028	115.23	Cultivar	Shaanxi	brown
Sru30	5.7510969	114.69	Cultivar	Yunnan	brown
Sru31	5.8846452	117.11	Cultivar	Shaanxi	brown
Sru32	5.6994126	113.27	Cultivar	Shaanxi	brown
Sru33	5.8452510	116.32	Cultivar	Shaanxi	brown
Sru34	5.8584156	116.66	Cultivar	Hebei	brown
Sru35	5.7443418	114.49	Cultivar	Shaanxi	brown
Sru36	5.8878756	117.28	Cultivar	Shaanxi	brown
Sru37	5.5336191	110.15	Cultivar	Shaanxi	brown
Sru38	5.9763822	119.16	Cultivar	Shaanxi	brown
Sru39	5.6468109	112.55	Cultivar	Hebei	brown
Sru40	5.7865203	115.05	Cultivar	Hebei	brown
Sru42	5.8858707	117.23	Cultivar	Fujian	brown
Sru44	5.9152776	117.69	Cultivar	Hubei	brown
Sru45	5.9668284	119.01	Cultivar	Hebei	brown
Sru46	5.5061244	109.12	Cultivar	Hebei	brown
Sru47	5.7764799	115.12	Wild	Shandong	brown
Sru48	4.9183146	95.88	Cultivar	Shandong	brown
Sru49	5.9916786	119.14	Cultivar	Shandong	brown
Sru50	5.6500371	112.37	Cultivar	Shandong	brown
Sru51	5.6395635	112.08	Cultivar	Hubei	brown
Sru52	5.5902006	111.54	Cultivar	Yunnan	brown
Sru53	5.9733798	118.83	Cultivar	Sichuan	brown
Sru54	5.5741188	105.36	Cultivar	Hunan	brown
Sru55	4.4327850	83.93	Cultivar	Shanxi	brown
Sru56	5.7086718	113.87	Cultivar	Gansu	brown
Sru57	4.9019262	97.74	Cultivar	Yunnan	brown
Sru58	5.5635774	111.02	Cultivar	Hebei	brown

## Data Availability

The original contributions presented in this study are included in the article/Supplementary Materials. Further inquiries can be directed to the corresponding author.

## Supplementary Materials

The following supporting information can be downloaded at: https://www.mdpi.com/article/10.3390/microorganisms13071685/s1, Figure S1: Geographic distribution of the 105 *Stropharia rugosoannulata* strains analyzed in this study. The map is color-coded to indicate the number of strains collected from each province in China. Table S1: Basic information of whole-genome sequenced 105 *Stropharia rugosoannulata* strains. This table lists essential details of the *Stropharia rugosoannulata* strains used in the study, including strain identifiers, origins (cultivated or wild), and sequencing statistics. Table S2: Detailed information of 83 core MNP marker-associated genes. Table S3: GS values of pairwise comparisons between all cultivars of *Stropharia rugosoannulata*.
