# Supplementary figures and images for: High-Resolution Core Gene-Associated Multiple Nucleotide Polymorphism (cgMNP) Markers for Strain Identification in the Wine Cap Mushroom Stropharia rugosoannulata
Source: Microorganisms. 2025 Jul 17;13(7):1685. doi: 10.3390/microorganisms13071685 (PMC12298363; doi:10.3390/microorganisms13071685)

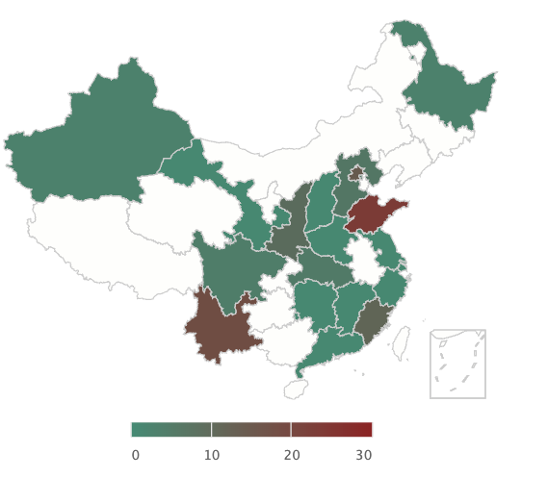

Supplement: Supplementary file 1 [file microorganisms-13-01685-s001.zip › microorganisms-3702243-supplementary.png]
